# Viroids as a Tool to Study RNA-Directed DNA Methylation in Plants

**DOI:** 10.3390/cells10051187

**Published:** 2021-05-13

**Authors:** Michael Wassenegger, Athanasios Dalakouras

**Affiliations:** 1 RLP AgroScience GmbH, 67435 Neustadt an der Weinstrasse, Germany; 2 Centre for Organismal Studies Heidelberg, University of Heidelberg, 69117 Heidelberg, Germany; 3 Hellenic Agricultural Organization Demeter, Institute of Industrial and Forage Crops, 41335 Larissa, Greece; nasosdal@gmail.com; 4 Hellenic Agricultural Organization Demeter, Institute of Plant Breeding and Genetic Resources, 57001 Thessaloniki, Greece

**Keywords:** viroids, RNA-directed DNA methylation, small RNAs, RNA interference, epigenetics, bisulfite sequencing

## Abstract

Viroids are plant pathogenic, circular, non-coding, single-stranded RNAs (ssRNAs). Members of the *Pospiviroidae* family replicate in the nucleus of plant cells through double-stranded RNA (dsRNA) intermediates, thus triggering the host’s RNA interference (RNAi) machinery. In plants, the two RNAi pillars are Post-Transcriptional Gene Silencing (PTGS) and RNA-directed DNA Methylation (RdDM), and the latter has the potential to trigger Transcriptional Gene Silencing (TGS). Over the last three decades, the employment of viroid-based systems has immensely contributed to our understanding of both of these RNAi facets. In this review, we highlight the role of *Pospiviroidae* in the discovery of RdDM, expound the gradual elucidation through the years of the diverse array of RdDM’s mechanistic details and propose a revised RdDM model based on the cumulative amount of evidence from viroid and non-viroid systems.

## 1. Viroids and RNAi

**Figure 1:**
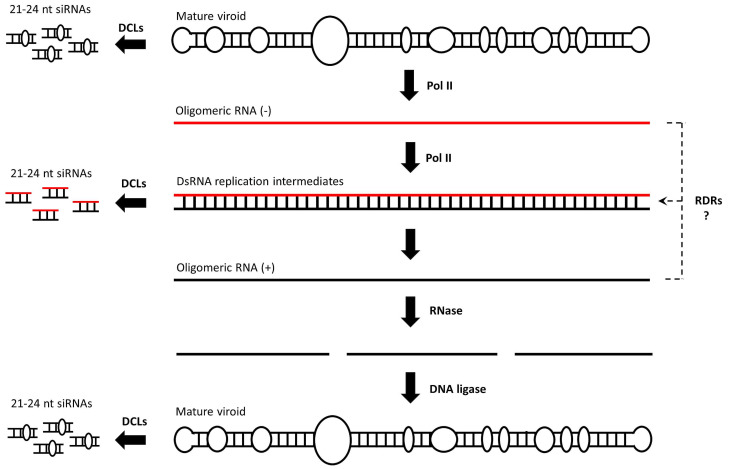
*Pospiviroidae* replicates through an asymmetric rolling circle mechanism. POL II is involved in all of the steps of viroid replication, generating dsRNA replication intermediates, but whether additional polymerases such as RDRs are also involved is not clear. DCLs may process both dsRNAs and the rod-shaped ssRNA mature molecule into siRNAs.

Viroids, the smallest known infectious agents, were discovered in 1971 by Theodor Diener [1]. Fifty years of ongoing research into these minimalistic pathogens has shed significant light on their biology. It is now well established that viroids are non-encapsidated, non-coding, circular ssRNAs, with a size of approximately 250–400 nucleotides (nt) and are classified into two major families, the *Pospiviroidae* and the *Avsunviroidae*, whose members replicate in the nucleus and the chloroplast, respectively [2,3,4,5]. The *Pospiviroidae* species, a potato spindle tuber viroid (PSTVd), has a rod-like structured 359-nucleotide genome that can be functionally and structurally divided into the following five domains: terminal left (TL), pathogenicity (P), central (C), variable (V) and terminal right (TR) [6,7]. When a PSTVd enters a plant cell, it is directed into the nucleus, most likely through the interaction of its TR domain with the host’s bromodomain-containing viroid RNA-binding protein 1 (VIRP1) [8,9]. In the nucleus, a PSTVd replicates via an asymmetric rolling circle mechanism [10]. A circular monomeric viroid (defined as having a plus polarity) is transcribed by a DNA-dependent RNA polymerase II (POL II) into linear oligomeric ssRNAs of a minus polarity [11]. These serve as a template for POL II, resulting in the accumulation of linear oligomeric ssRNAs of a plus polarity. The latter is cleaved by a type III RNase into fragments that are finally ligated by DNA ligase 1 into unit length circular RNAs [12] (Figure 1). A mature viroid then exits the nucleus into the cytoplasm, moves to neighboring cells through plasmodesmata and then to distant parts of the plant through the phloem [13,14]. The most important features of viroids that make them a very specific and unique pathogen are the fact that they do not encode for proteins and that their infection cycle does not involve DNA intermediates, although their RNAs are produced by POL II, an enzyme that generally transcribes DNA.

It has been suggested that dsRNAs are generated during viroid replication [15,16,17]. The source of these dsRNAs is not clear, but they are most probably generated upon POL II transcription of an oligomeric plus from an oligomeric minus transcript and their kinetic interaction. Whether the RNA-directed RNA polymerase (RDR) transcription of these oligomeric transcripts or even of the mature viroid contributes to dsRNA generation is not clear but cannot be excluded. In any case, viroid replication triggers the host’s RNAi mechanism [18,19,20]. At least in *Nicotiana benthamiana*, all four dicer-like endonucleases (DCLs) seem to process a PSTVd into a small interfering RNA (siRNA) [21,22]. It is most likely that dsRNA replication intermediates are preferentially processed by DCL2/3/4 into 21–24-nucleotide siRNAs, but it cannot be ruled out that a microRNA (miRNA) precursor-like, rod-shaped, mature viroid is also processed by a DCL1 into 21-nucleotide siRNAs, although in the latter case the occurring siRNAs would contain bulges (Figure 1). Whatever their source may be, DCL4-produced 21-nucleotide siRNAs are generally loaded onto Argonaute 1 (AGO1), leading to the formation of an RNA-induced silencing complex (RISC) [23]. An RISC recognizes the complementary transcripts for cleavage in a process termed PTGS [24]. DCL2-produced 22-nucleotide siRNAs are also loaded onto AGO1 and are suggested to change the conformation of AGO1. This alteration is assumed to affect the target RNA degradation. Thus, instead of RNA cleavage, 22-nucleotide siRNA-containing AGO1 complexes seem to either recruit an RDR6 to initiate transitivity and secondary siRNA formation or to inhibit the translation of the targeted mRNA [25,26]. Finally, DCL3-produced 24-nucleotide siRNAs are predominantly loaded onto AGO4 and are involved in the RdDM of cognate sequences [27]. Viroid siRNAs are 5′-phosphorylated and 3′-methylated, as all plant siRNAs are [28]. Small RNA deep sequencing revealed that viroid siRNAs are of both polarities and map preferentially, in the same way virus- and transgene-derived siRNAs do, to specific hotspot regions [29]. At least the 21-nucleotide viroid siRNAs are biologically active, since they trigger the degradation of mRNA’s containing regions that are complementary to viroid siRNAs [30,31]. Interestingly, the mature circular viroid is considered to be resistant to RISC-mediated degradation, most likely due to its extensive secondary structure [29]. Nevertheless, tomato plants expressing a hairpin PSTVd RNA transgene construct were found to be resistant to viroid infection, indicating that the RNAi machinery could target PSTVd-specific RNA molecules that are essential for viroid infection [32].

## 2. RNA-Directed DNA Methylation

**Figure 2:**
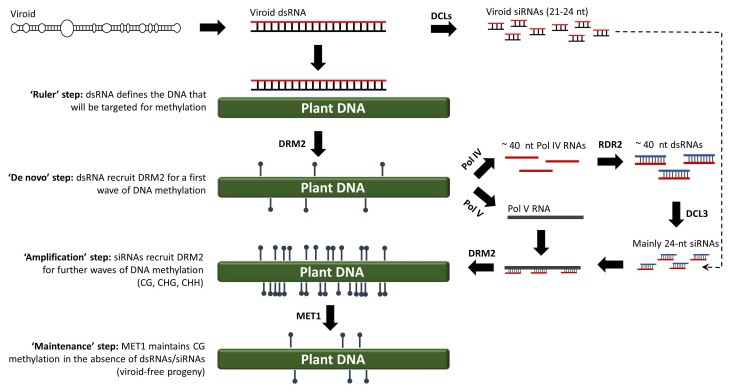
An alternative RdDM model for both viroid and non-viroid systems, wherein the very first de novo methylation step is triggered by dsRNAs rather than siRNAs.

In eukaryotes, DNA methylation refers to the addition of a methyl group to the fifth atom in the six-atom ring of cytosine residues (Cs). As a chemical modification, DNA methylation was discovered in 1948 by Rollin Hotchkiss, almost simultaneous to the identification of DNA as genetic material by Avery, MacLeold and McCarty. Almost thirty years later, in 1975, Holliday and Pugh proposed that DNA methylation is an important epigenetic mark [33]. However, the mechanism by which DNA methylation was induced remained elusive for many years. In plants, DNA methylation was widely considered to be mediated by DNA–DNA interactions. However, in 1994, a study using viroid-infected tobacco plants showed that de novo DNA methylation is mediated by RNA molecules, thus aptly termed ‘RNA-directed DNA methylation’ (RdDM) [34]. Today, almost 30 years after the RdDM discovery, the widely accepted model suggests that 24-nucleotide siRNAs (canonical RdDM) or, in exceptional cases, 21/22-nucleotide siRNAs (non-canonical RdDM), are loaded onto AGO4 and guide domains-rearranged methyltransferase 2 (DRM2) to methylate cognate DNA, most likely through a process wherein siRNAs directly interact with DNA or interact with the nascent transcripts produced by RNA polymerase V (POL V) [35,36,37]. However, a growing body of evidence challenges the validity of this model. To begin with, it has been suggested that POL V is recruited to an already methylated DNA template, thus it can hardly be involved in the very first step of de novo methylation of a completely unmethylated DNA [38]. More importantly, RdDM is not eliminated in an *Arabidopsis thaliana* quadruple *dcl1 dcl2 dcl3 dcl4* mutant, suggesting that DCL-produced siRNAs are dispensable for RdDM [39]. In addition, AGO4, which is thought to be involved in both canonical and non-canonical RdDM, is not always required for RdDM. AGO4 contains the DDH motif and has a slicer activity [40]. During sense RdDM (S-RdDM), a single-stranded RNA is cleaved by the siRNAs loaded onto AGO4 and the cleaved transcripts are copied by RDRs into dsRNAs [40]. However, during inverted repeat RdDM (IR-RdDM), the generation of a dsRNA would not rely on AGO4 activity, but on the mere transcription of hairpin RNA-producing DNA. Indeed, AGO4 is not required in IR-RdDM, at least not in *Arabidopsis thaliana* [41]. Collectively, these data suggest that RdDM is triggered not by siRNAs, but by long dsRNAs (>50 bp) [42,43,44,45]. According to our model, long dsRNAs appear to define the DNA region that will be methylated in a ruler-like fashion (Step 1, Figure 2). The dsRNA–DNA interaction may involve the formation of triple helices, as has been shown to take place in mammals between long non-coding RNAs (lncRNAs) and DNA [46]. Such aberrant structures could attract DRM2 to impose a first (perhaps incomplete) wave of de novo methylation on fully unmethylated DNA (Step 2, Figure 2). The resulting (and incompletely) methylated DNA seems to recruit POL IV and POL V. POL IV generates short transcripts (40–50 nt) that are copied by RDR2 into 40–50 bp dsRNAs that are cleaved by DCL3 into 24-nucleotide siRNAs [47]. The AGO4-loaded 24-nucleotide siRNAs will now hybridize the newly produced POL V transcript, and this re-initiates the recruitment of DRM2 to impose a second wave of methylation marks (Step 3, Figure 2) [48,49]. As siRNAs are amplified by this self-reinforcing loop mechanism, methylation will sequentially increase until nearly all of the Cs are methylated to CG, CHG and CHH sequence contexts in both of the DNA strands [50]. Importantly, upon cell division and in the absence of dsRNAs/siRNAs, CG methylation can be maintained through the action of methyltransferase 1 (MET1) (Step 4, Figure 2) [51]. To a lesser extent, CHG methylation will be maintained by chromomethylase 3 (CMT3) [52]. However, CHH methylation cannot be maintained, as it always needs to be de novo established and requires the presence of dsRNAs/siRNAs [45].

Over the last 30 years, the role of viroid-based systems in the elucidation of the RdDM mechanism and in refining corresponding models has been undisputable. Based on our current knowledge of RdDM and the above model, in the following, we will retrospectively navigate through the various stations of viroid-based RdDM, from 1994 to today.

## 3. Discovery of RdDM in Viroid-Infected Plants

**Figure 3:**
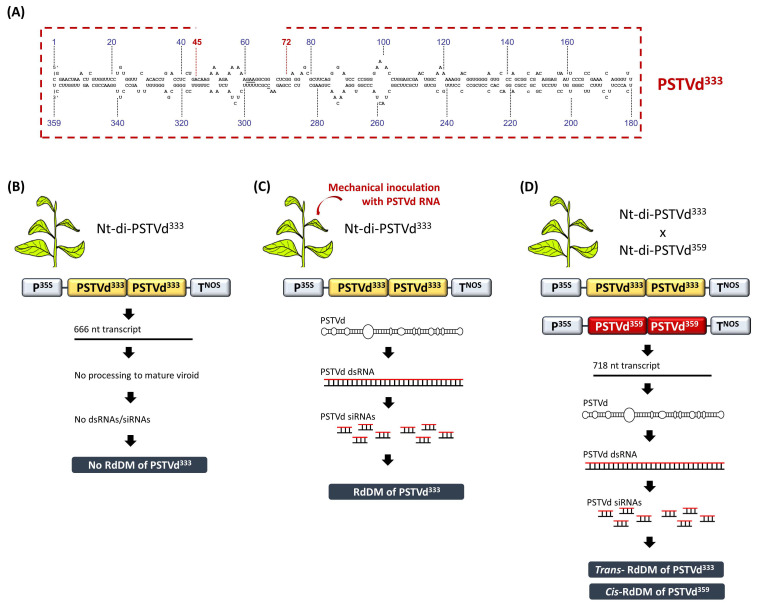
The discovery of RdDM in viroid-infected tobacco plants. (**A**) Schematic representation of the PSTVd^333^ containing a 26-nucleotide deletion compared to a full length PSTVd^359^. (**B**) Nt-di-PSTVd^333^ plants are free of methylation. (**C**) Upon viroid infection, Nt-di-PSTVd^333^ plants exhibit dense RdDM. (**D**) The crossing of Nt-di-PSTVd^333^ with Nt-di-PSTVd^359^ induced both cis and trans RdDM in CG, CHG and CHH contexts. Upon the segregation of the PSTVd^359^ infectious transgene, the PSTVd^333^ progeny maintains CG methylation for at least two generations.

RdDM was discovered in a study involving viroid-infected tobacco plants [34]. In that study, transgenic tobacco plants carried an expression cassette containing three head-to-tail linked, plus-oriented, full length PSTVd^359^ cDNA copies (Nt-tri-PSTVd^359^). The transcription of this transgene construct led to the formation of mature, circular and infectious PSTVd molecules. However, at that time it was not known that replicating PSTVd and the concomitant accumulation of viroid dsRNAs/siRNAs could lead to the DNA methylation of the cognate sequences. Thus, it was puzzling that the PSTVd^359^ cDNA proved severely resistant to methylation-sensitive restriction endonucleases when genotyping the transgenic lines using Southern blot analysis. In order to exclude the possibility that methylation was induced by the DNA–DNA interactions, transgenic tobacco plants were generated expressing a head-to-tail linked dimeric PSTVd^333^ cDNA (Nt-di-PSTVd^333^), where each PSTVd^333^ fragment carried a 26-bp deletion compared to the full length PSTVd^359^ (Figure 3A). Upon transcription of the dimeric PSTVd^333^, a 666-nucleotide RNA was produced but, due to the 26-nucleotide deletion, it could not be processed into a mature PSTVd. Thus, these transgenic lines were not PSTVd-infected. Moreover, no methylation of the PSTVd^333^ was detected, suggesting that the agent of DNA methylation is something other than the mere DNA–DNA interactions that were suggested to occur upon the presence of tandem cDNA repeats (Figure 3B). Indeed, when the Nt-di-PSTVd^333^ plants were mechanically-infected with PSTVd RNA, the PSTVd^333^ cDNA exhibited dense methylation, demonstrating that an RNA agent directed the DNA methylation in the cognate DNA sequences (Figure 3C) [34].

## 4. CHH Is the Hallmark of the RNA-Directed de novo DNA Methylation

Further studies on viroid-based systems provided important insights into the RdDM mechanism. In particular, the sequence context of the methylated Cs was elucidated by bisulfite sequencing. In PSTVd-infected Nt-di-PSTVd^333^ plants, both DNA strands of the PSTVd^333^ sequences were equally methylated [50]. Moreover, in both strands, the Cs of any sequence context (CG, CHG or CHH) were methylated. This finding was in stark contrast to the situation of mammalian systems where C methylation is predominantly found in a CG context [53]. In plants, we now know that, whereas CG and CHG methylation can be maintained even in the absence of dsRNAs/siRNAs using MET1 and CMT3, respectively, CHH methylation always needs to be re-established in an RNA-dependent manner, and thus is a hallmark of RdDM [54]. Interestingly, the methylation was very dense in the full body of the PSTVd^333^ cDNA, with the exception of the region flanking the 26-nucleotide deletion (compared to PSTVd^359^), most likely due to the perturbation of the viroid dsRNA with the PSTVd^333^ cDNA at that site [50].

## 5. CG Methylation Can Be Maintained in the Absence of dsRNAs/siRNAs

In order to analyze the trans-generational maintenance of viroid-induced RdDM, the Nt-di-PSTVd^333^ plants were crossed with transgenic tobacco plants carrying two head-to-tail linked, minus-oriented, full length PSTVd^359^ cDNA copies (Nt-di-PSTVd^359^), with the latter allowing for the occurring transcript to be processed into a mature, infectious viroid (Figure 3D) [55]. The generated dsRNAs/siRNAs not only triggered the degradation of the 666-nucleotide transcript, but also directed the DNA methylation in both the PSTVd^359^ cDNA (cis-RdDM) and the PSTVd^333^ cDNA (trans-RdDM) (Figure 3D). Bisulfite sequencing revealed that cis-RdDM and trans-RdDM were involved, as expected, in the methylation of Cs in all sequence contexts (CG, CHG and CHH) [48]. In the viroid-free progeny (segregation of the PSTVd^359^ cDNA transgene), PSTVd^333^ cDNA lost all CHG and CHH methylation, but stably maintained CG methylation, for at least up to two consecutive generations. Chromatin immunoprecipitation analysis showed that the maintenance of CG methylation was not associated with an increase in the dimethylation of histone H3 lysine 9 or a decrease in the acetylation of H3 [55]. These data were in contrast to previous studies that suggested that CG methylation could be maintained in promoter sequences but not in gene bodies [56].Yet, they were in agreement with other studies that showed the widespread maintenance of CG methylation in gene bodies [57,58]. The role of CG methylation in gene bodies is not very clear, but it does not seem to have a negative impact on the transcription [59]. Indeed, in the viroid-free Nt-di-PSTVd^333^ post-segregation progeny that contained CG methylation, the 666-nucleotide transcript accumulated to the levels that had been found before the viroid infection [55].

## 6. 30 bp Is the Minimum Target Size for RdDM

**Figure 4:**
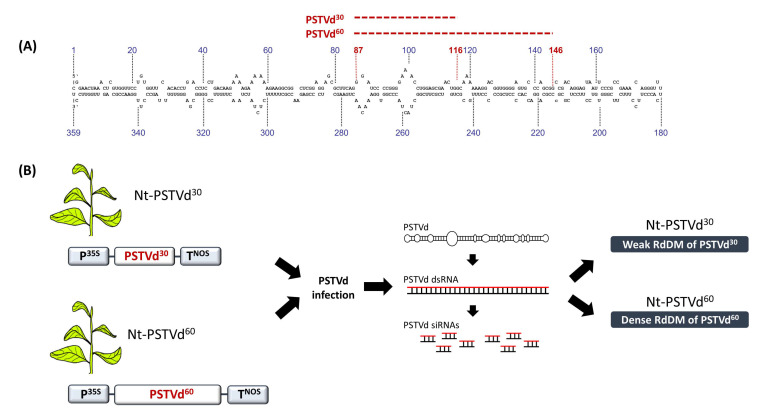
Minimum target size for RdDM. (**A**) Schematic representation of the PSTVd^30^ and PSTVd^60^ sequences. (**B**) Viroid infection in Nt-PSTVd^30^ and Nt-PSTVd^60^ plants leads to an efficient RdDM only on the PSTVd^60^ transgene.

To investigate the minimum target size for RdDM, transgenic tobacco plants carrying non-infectious PSTVd cDNA subfragments of 30 and 60 bp in size (Nt-PSTVd^30^, Nt-PSTVd^60^) were generated and PSTVd-infected by mechanical inoculation (Figure 4A,B) [60]. In the infected plants, the methylation status of each transgene was analyzed using bisulfite sequencing. While PSTVd^60^ cDNA was densely methylated, PSTVd^30^ was merely methylated, suggesting that approximately 30 bp is the minimum target size for RdDM initiation (Figure 4B) [60]. It needs to be highlighted that the same 30 bp region that was poorly methylated in PSTVd^30^, was densely methylated in PSTVd^60^. If siRNAs triggered the RdDM, then a given 24-nucleotide siRNA could not possibly discriminate between the corresponding 24 bp region in PSTVd^60^ and in PSTV^30^ and thus could, in principle, impose methylation marks with equal efficiency on both transgenes. However, this did not happen. Based on these observations, we favor the scenario in which the initial interaction for RdDM was between a large dsRNA (>50 bp) and the DNA. In the case of PSTVd^60^, this interaction managed to exceed a certain kinetic/thermodynamic threshold to recruit DRM2, whereas in the case of PSTVd^30^ this might not have been achieved. Indeed, such a threshold mechanism should have been evolutionarily maintained in order to avoid off-target RdDM events that could negatively affect plant fitness.

## 7. Local DNA Features Inhibit RdDM

**Figure 5:**
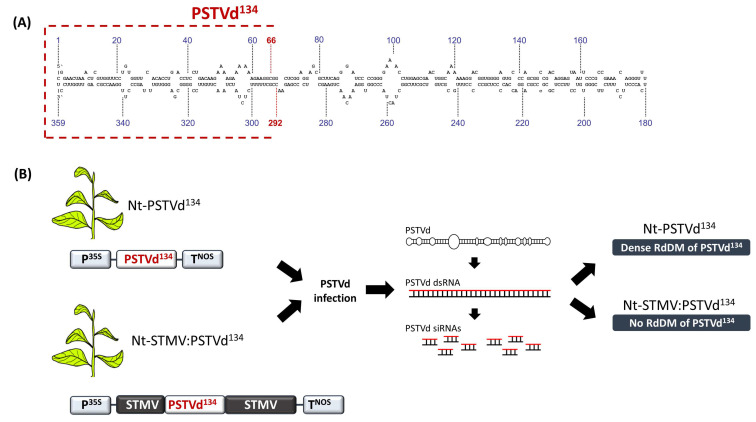
RdDM is affected by local DNA features. (**A**) Schematic representation of the PSTVd^134^. (**B**) Viroid infection in Nt-PSTVd^134^ and Nt-STMV:PSTVd^134^ leads to an efficient RdDM of the PSTVd^134^ fragment only in the case of Nt-PSTVd^134^ plants.

The aforementioned threshold level does not seem to be solely influenced by the extent of base pair interactions between the DNA and the dsRNA, but also by the inherent structure of the DNA itself. Indeed, upon the PSTVd infection of the tobacco plants carrying a 134-bp PSTVd cDNA subfragment (PSTVd^134^) that was inserted into a chimeric satellite of the tobacco mosaic virus’ (STMV) cDNA construct (STMV:PSTVd^134^), the PSTVd^134^ was inefficiently targeted for RdDM [61]. In contrast, the same PSTVd^134^ sequence became heavily methylated when it was not flanked by satellite cDNA sequences (Figure 5). Replicating and non-replicating satellite sequences are known to exhibit complex secondary structures that may render them resilient to RNAi [20]. Drawing an analogy, one may hypothesize that the complex secondary structures of the targeted DNA (or its nascent transcript) exhibited insignificant thermodynamical and/or kinetical interactions with the de novo methylation-directing RNA molecules. Supporting our hypothesis, a study in *A. thaliana* reported that RdDM efficiency is heavily influenced by local DNA features [62].

## 8. RdDM Is Not Necessarily Coupled to PTGS and Transitivity

**Figure 6:**
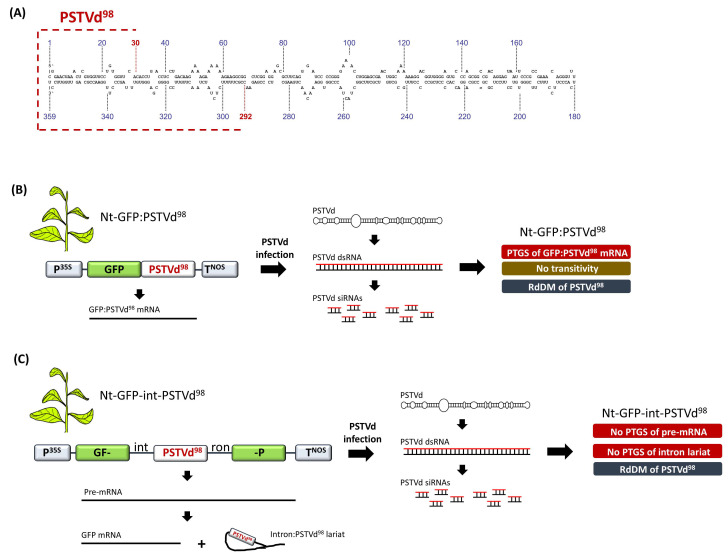
RdDM is uncoupled with transitivity and PTGS. (**A**) Schematic representation of the PSTVd^98^ sequence. (**B**) Viroid-infection in Nt-GFP:PSTVd^98^ and (**C**) Nt-GFP-int-PSTVd^98^ leads to the efficient RdDM of the PSTVd^98^ fragment in both cases, despite the absence of transitivity (case Nt-GFP:PSTVd^98^) and PTGS (case Nt-GFP-int-PSTVd^98^).

In transcribed sequences, RdDM and PTGS appear to be tightly connected. Both processes are triggered by dsRNAs and in all examples of PTGS where methylation studies were included, the PTGS target regions were found to be also de novo methylated [63]. However, in the case of the RdDM of non-transcribed sequences (e.g., promoters), this general rule does not seem to apply. In the case of the RNAi of coding regions, primary dsRNAs will be processed into primary siRNAs. Thus, PTGS/degradation of mRNAs will be induced. Under certain conditions (e.g., the presence of 22-nucleotide siRNAs and/or the absence of introns in the gene of the target transcript), PTGS may also involve the initiation of RDR6-mediated transitivity and thus the production of secondary dsRNAs and secondary siRNAs [64,65]. It is most likely that secondary dsRNAs and siRNAs contribute towards RdDM and PTGS, respectively [66]. Moreover, they seem to be indispensable for the establishment of systemic silencing [67]. However, it is unlikely that they are essential for the initiation of RdDM. When tobacco plants carrying a transgene composed of the PSTVd^98^ fragment fused to the 3′ of the GFP coding region (Nt-GFP:PSTVd^98^) were infected with PSTVd, viroid siRNAs mediated PTGS/degradation of the GFP:PSTVd^98^ mRNA (Figure 6A,B) [31]. Interestingly, siRNA analysis of Nt-GFP:PSTVd^98^ revealed that no RDR6-mediated transitivity of the GFP was detected, suggesting that the structure of PSTVd^98^ inhibited RDR6 activity. However, despite the absence of transitivity, the PSTVd^98^ cDNA fragment (but not the GFP cDNA) was densely methylated, suggesting that it was the double stranded viroid replication intermediates, and not the dsRNAs derived from transitivity, that guided the RdDM (Figure 6B) [31].

In a complementary study, the PSTVd^98^ was inserted into an artificial intron of the GFP (GFP-int-PSTVd^98^), and the generated construct was stably transformed into tobacco (Nt-GFP-int-PSTVd^98^) (Figure 6C) [68]. In these plants, transgene transcription would lead to the formation of a pre-mRNA and, upon splicing, to a mature GFP mRNA as well as a spliced intronic lariat containing the PSTVd^98^ that would be retained in the nucleus. The PSTVd-infection of Nt-GFP-int-PSTVd^98^ resulted in an abundant accumulation of PSTVd siRNAs that, nevertheless, failed to trigger pre-mRNA or intronic lariat RNA degradation [68]. Indeed, PTGS is a cytoplasmic process, and only a few reports have suggested nuclear PTGS [69]. Nevertheless, despite the absence of PTGS, the PSTVd^98^ fragment of the GFP-int-PSTVd^98^ cDNA was densely methylated (Figure 6C) [68]. Collectively, these data demonstrate that PTGS and transitivity are not a prerequisite for RdDM in gene bodies.

## 9. RdDM Efficiency Depends on Sequence Identity between Trigger RNA and Target DNA

**Figure 7:**
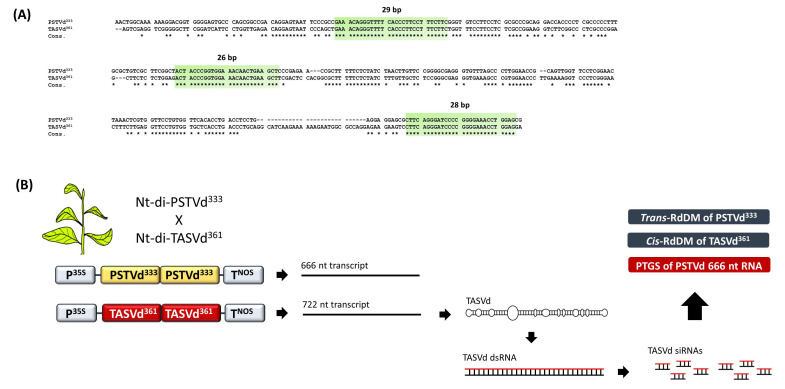
RdDM is influenced by trigger–target sequence identity. (**A**) Alignment of PSTVd^333^ and TASVd^361^, showing the three regions of >24 nt sequence identity. Asterisks denote sequence consensus. (**B**) Crossing of Nt-di-TASVd^361^ with Nt-di-PSTVd^333^ leads to an abundant accumulation of TASVd siRNAs that map onto only three PSTVd^333^ sites. However, PSTVd^333^ cDNA was methylated almost at its full size, despite the absence of siRNAs.

In all of the above cases, PSTVd transgene constructs were used as RdDM targets and PSTVd infection was used as a RdDM trigger. The extent to which an infection from another member of *Pospiviroidae* would induce RdDM in PSTVd transgenes had not been analyzed. To investigate the potential of a PSTVd-related viroid to mediate RdDM of a PSTVd cDNA, a tomato apical stunt viroid (TASVd) was used as a RdDM trigger. A PSTVd and a TASVd share 81% of their sequence identity and exhibit only three regions (26, 28 and 29 bp) of continuous sequence identity (Figure 7A). This provided an excellent opportunity to investigate the role of siRNAs in RdDM, since TASVd siRNAs would only map perfectly onto these three PSTVd^333^ regions and thus, if siRNAs were the actual RdDM triggers, would methylate only these three regions. A head-to-tail, full length and infectious TASVd^361^ dimer transgene was used to transform the tobacco plants (Nt-di-TASVd^361^) [70]. Next, Nt-di-TASVd^361^ was crossed with Nt-di-PSTVd^333^. In the progeny containing both transgenes, a TASVd infection resulted in a very efficient cis-RdDM of the TASVd^361^ cDNA, but also in the significant trans-RdDM of the PSTVd^333^ cDNA (Figure 7B). Small RNA deep sequencing revealed that the TASVd siRNAs mapped onto the full length of the TASVd^361^ cDNA, but only in three (26, 28 and 29 bp) regions in the PSTVd^333^ cDNA. Nevertheless, moderate methylation of the full length PSTVd^333^ was detected, strongly suggesting that the trans-RdDM on the PSTVd^333^ cDNA was induced by the TASVd dsRNA molecules rather than the TASVd siRNAs [70]. Obviously, the 81% of the sequence identity shared by the TASVd^361^ RNA and the PSTVd^333^ cDNA proved to be sufficient to trigger the RdDM, albeit not as efficient as the RdDM observed in TASVd^361^ cDNA, which shared 100% of its sequence identity with the TASVd^361^ RNA.

## 10. Future Perspectives

From the above examples, it is evident that viroid-based systems have greatly contributed to the elucidation of RdDM in plants and will most likely continue to do so in the future. Yet, several issues remain to be investigated. At least some of the viroid-induced symptoms in plants seem to be the outcome of a viroid siRNA-mediated cleavage of endogenous mRNAs [71,72,73]. Whether the viroid-induced symptoms are also related to the viroid-induced epigenetic changes remains to be established. Accordingly, comprehensive studies involving whole genome bisulfite sequencing coupled with the chromatin immunoprecipitation sequencing of DNA from viroid-infected plants and the transcriptome analyses of RNA from symptomatic and symptomless viroid-infected plants would be immensely insightful. Indeed, at least in cucumber, hop stunt viroid infection affects the DNA methylation of the host ribosomal RNA genes [74]. In tomato plants, PSTVd siRNAs match partially or fully with endogenous mRNAs and lead to their degradation [30]. However, the small sequence identity of less than 30 bp may not be enough to mediate RdDM, as discussed above in relation to the minimum target size requirements for RdDM [60]. The presence of plant DNA sequences having a minumum size of 30 bp and exhibiting at least 70–80% sequence identity with a given viroid need to be examined. Such regions could be candidates for viroid-induced RdDM.

Finally, another interesting topic that has not been investigated yet, is whether members of the *Avsunviroidae* can also trigger RdDM. Of course, these viroids replicate in the chloroplast, where no RNAi/RdDM occurs [75]. However, when they enter the plant cell, it is thought that they are first transported into the nucleus before being targeted to the chloroplasts [76]. In this context, it should be noted that an *Avsunviroidae* infection is associated with the generation of abundant viroid siRNAs [77]. Eggplant latent viroid (ELVd), a member of the *Avsunviroidae*, has only been shown to infect eggplant, the species in which it was discovered [78]. Nevertheless, our preliminary data suggest that in tobacco plants (a non-natural host for ELVd) transformed with a theoretically infectious head-to-tail trimeric cDNA (ELVd^333^), RdDM of the cognate ELVd^333^ cDNA in the nucleus can take place. Future analysis should demonstrate whether chloroplast-replicating viroids can indeed trigger the RdDM of nuclear sequences.
